# Supplementary material for: Determinants of farmers’ decision-making behavior for black soil protection in Northeast China—A case of Jilin Province*
Source: PLoS One. 2025 Dec 19;20(12):e0339285. doi: 10.1371/journal.pone.0339285 (PMC12716778; doi:10.1371/journal.pone.0339285)
Supplement: Attachment — Submitted filename: Response_to_Reviewers.docx [file pone.0339285.s002.docx]

Response to the Reviewers’ Comments

We thank the reviewers for taking the time to read and give their comprehensive and constructive comments, which have improved our manuscript. Below, we provide a point-by-point response to your comments and suggestions and how each one has been addressed in the revision.

Response to Reviewer 1 Comments

**Comment 1**. The research questions are not clearly formalised: they do not emerge explicitly in either the introduction or the objectives section. It is essential to make them clear, explicit and testable, especially since this is an SEM model.

**Response:** We are sorry for the shortcomings in our previous manuscript. The bibliography recommended by reviewers is of great help to the manuscript and helps the author to have a deeper understanding of the existing research. In the abstract part, we have made some modifications, and at the same time, we have written the clear research questions of this paper in the penultimate paragraph of the introduction.

**Comment 2**. The originality is only partial. The application of TPB to agricultural behaviour is now widespread: there is no strong justification for the innovative contribution compared to the extensive existing literature.

**Response:** We thank the reviewer for this comment. We have made relevant modifications in the penultimate paragraph of the introduction of the article, which fully illustrates the innovation of this research.

**Comment 3**. The literature is largely self-referential and not up to date with recent contributions on the adoption of sustainable agricultural practices at the international level.

**Response:** We thank the reviewer for this comment. In the third paragraph of our introduction, we added sustainable practices for black soil conservation in the United States and Ukraine.

**Comment 4**. The limitations of the TPB are not critically discussed, for example, the lack of attention to the institutional context and structural inequalities.

**Response:** We thank the reviewer for this comment. In the penultimate paragraph of the introduction, we have given a detailed answer to this question.

**Comment 5**. The use of SEM is technically appropriate for testing models with latent variables and multiple relationships. Reliability (Cronbach's α), convergent validity (AVE) and discriminant validity tests are correctly implemented. However, the sampling process is weak and it is not explained transparently how the 486 farmers were selected. Recruitment via online platforms does not guarantee representativeness.

**Response:** First of all, I would like to thank the reviewers for their approval of the model, and thank you for your questions. In response to your question, we detailed the extraction of the survey in the third paragraph of Materials and Methods in the article.

**Comment 6**. The justification for the study area (Jilin) is broad but poorly connected to theoretical or comparative reasoning. There is no reflection on how generalisable the results are outside this context.

**Response:** We thank the reviewer for this comment. In the third paragraph of Materials and Methods in the article, we focused on the reasons for selecting Jilin Province, because Jilin Province in China is a typical area for black soil protection, and the "pear tree model" is also promoted throughout the country.

**Comment 7**. The SEM formulas are reported correctly, but the text does not clearly illustrate how the standardised weights were interpreted.

**Response:** We thank the reviewer for this comment. We illustrate the means and standard deviations of the selected variables in Table 2, and verify the reliability of the scale in the first paragraph of the results, which indicate good internal consistency. So no normalized weights are reported.

**Comment 8**. The results are consistent with the TPB literature: behavioural intention mediates the effect of cognitive and normative variables on actual behaviour. However, the tables are numerous and redundant. The information on AVE, CR, and CFI could be better summarised. The figures are descriptive and do not add interpretative substance to the model.

**Response:** We thank the reviewer for these comments and affirmation of the manuscript. What we want to explain is that the AVE, CR, and CFI you mentioned are not in the same table. In the structural equation model, CFI measures the fitting degree of the CFA model. AVE and CR will be calculated only on the premise that the fitting degree passes. AVE and CR test the convergence validity and combined reliability of each dimension of the scale, so we did not put the three indicators you mentioned in one table. Thank you for your understanding.

**Comment 9**. The standardised estimates report limited effects: for example, the contribution of subjective norms is statistically weak (β=0.098).

**Response:** We thank the reviewer for this comment. We explain the reasons for the weak significance of the subjective norm in the third paragraph in the SEM results and refer to three articles of literature.

**Comment 10**. There is a lack of sensitivity analysis or robustness testing of the model.

**Response:** We thank the reviewer for this comment. We explain this in the last paragraph of our conclusion in the article, thank you for your understanding.

**Comment 11**. The discussion highlights the crucial role of training, institutional communication and economic incentives in promoting protective behaviour. However, this remains too general and lacks a real comparison with international literature or similar contexts (e.g. other countries with soil degradation problems).

**Response:** We thank the reviewer for this comment. In the third paragraph of the introductory part of the article, we introduce the practices of black land protection in the United States and Ukraine.

**Comment 12**. Policy implications are stated but not supported by concrete scenarios or simulations.

**Response:** We thank the reviewer for this comment. We have made corresponding changes in the conclusions

**Comment 13**. Methodological limitations are only mentioned marginally (absence of causality, risk of bias in questionnaires, etc.), and future research developments are vague.

**Response:** We thank the reviewer for this comment. We revised this in the conclusion part, and put forward the future research development.

Response to Reviewer 2 Comments

**Comment 1**: Remove Cronbach’s alpha from Table 3.

**Response:** We thank the reviewer for this comment. Changes have been made as you suggested.

**Comment 2**: The lack of comparative analysis with findings from other related studies. It is necessary to discuss research that has obtained similar or contrasting results and explain the reasons for these similarities and differences.

**Response:** We thank the reviewer for this comment. According to your question, we explain the reason why the subjective norm is weakly significant in the third paragraph of SEM results, and refer to three literatures.

Comment 3: The following articles should be cited in the paper:

**Response:** We thank the reviewer for this comment. We have already referenced both articles in our article.
