# Supplementary material for: Determinants of farmers’ decision-making behavior for black soil protection in Northeast China—A case of Jilin Province*
Source: PLoS One. 2025 Dec 19;20(12):e0339285. doi: 10.1371/journal.pone.0339285 (PMC12716778; doi:10.1371/journal.pone.0339285)
Supplement: Attachment — Submitted filename: Response_to_Reviewers_auresp_2.docx [file pone.0339285.s003.docx]

Response to the Reviewers’ Comments

We thank the reviewers for taking the time to read and give their comprehensive and constructive comments, which have improved our manuscript. Below, we provide a point-by-point response to your comments and suggestions and how each one has been addressed in the revision.

Response to Reviewer 1 Comments

**Comment 1**. In the introduction (lines 28-41), write, 'As one of the three black soil areas in the northern hemisphere, north-eastern China is an important area for grain production… It takes 200-400 years to generate 1 cm of black soil’. I recommend separating the geographical part from the agronomic part.

**Response:** We have made the changes as you suggested and thank you very much for your suggestions. We didn't separate before because we wanted to explain the importance of black soil in China. After all, China is a big agricultural country. At the same time, food production and land are inseparable.

**Comment 2**. Lines 39-48: "However, due to long-term intensive use... improve overall food production capacity". Here, three problems ("thin-lean-hard") are described: a bulleted list or shorter sentences would be better.

**Response:** We thank the reviewer for this comment. We have not revised your modification suggestion. First of all, we have briefly introduced it when explaining "thin-lean-hard", and each sentence cites the corresponding literature. The reason why we didn't make a table is that the current situation of black soil has undergone long-term changes, and not all these data are public. We can only find out the current situation of black soil. Please understand.

**Comment 3**. In Discussion/Interpretative results, lines 349-369: "Perceptual control of behaviour is a factor influencing behavioural intention... awareness of investment and use of funds for black soil protection (PBC3)". Too dense: a summary and the use of specific references are suggested.

**Response:** We thank the reviewer for this comment. We have revised your suggestion, deleted some contents, and cited relevant literature.

**Comment 4**. Lines 382-389: "in general, attention should be paid to the mutual influence of farmers" cognition. promoting farmers' participation in black soil protection.’ This repeats the concept already expressed above (covariance of constructs).

**Response:** We thank the reviewer for this comment. According to your suggestion, we have made modifications to supplement the novelty of the planned behavior theory for farmers' black soil protection behavior.
